# The Impact of the COVID-19 Pandemic on Forensic Psychiatric Examination

**DOI:** 10.3390/diagnostics15040483

**Published:** 2025-02-17

**Authors:** Daniela Margareta Varga, Florica Voiță-Mekeres, Camelia Liana Buhaș, Gabriel Mihai Mekeres, Florina Madălina Mîndru, Nuțu Cristian Voiță, Lavinia Davidescu

**Affiliations:** 1 Doctoral School of Biomedical Sciences, University of Oradea, 410073 Oradea, Romania; varga.danielamargareta@student.ro (D.M.V.); cameliabuhas@uoradea.ro (C.L.B.); mindru.florinamadalina@student.uoradea.ro (F.M.M.); 2 County Clinical Emergency Hospital of Oradea, 410087 Oradea, Romania; mekeres.gabriel.mihai@student.uoradea.ro; 3 Department of Morphological Disciplines, Faculty of Medicine and Pharmacy, University of Oradea, 410073 Oradea, Romania; 4 Medical Department, Faculty of Medicine and Pharmacy, University of Oradea, Universitatii Street Nr.1, 410087 Oradea, Romania; lavinia.davidescu@didactic.uoradea.ro; 5 County Emergency Hospital Cluj-Napoca, 400000 Cluj-Napoca, Romania; voita_cristi@yahoo.com

**Keywords:** forensic psychiatric examination, COVID-19, discernment

## Abstract

**Background/Objectives:** Forensic psychiatric expertise significantly contributes to clinical criminology. Interdisciplinary investigations, evaluation tactics, and the use of criminology indices are essential for psychosocial prognostic assessments of conflict, aggression, adaptability, and recovery. **Methods:** This study aims to assess the impact of the COVID-19 pandemic on forensic psychiatric expertise by analyzing demographic variables such as age, gender, background, and other relevant data including criminal records, forensic antecedents, personal pathological antecedents, diagnosis, and discernment. Our study included 186 patients categorized into pandemic and post-pandemic periods. **Results:** Most participants were male, with no significant rural–urban distribution differences. During the COVID period, common criminal acts included aggression and child pornography, followed by attempted murder. A significant association was found between discernment and psychiatric disorders (*p* < 0.0011), with 67.6% of the patients lacking discernment having mental illnesses. Legal outcomes varied with discernment; 60.3% of patients without discernment required mandatory hospitalization (*p* < 0.0011). **Conclusions:** Medico-legal antecedents were more frequent during the pandemic, and safety measures were more commonly applied to those lacking discernment.

## 1. Introduction

Legal medicine and psychiatry are specialties involved in the protection of public health through their particular implications for general pathology, the specific methods used for solving their objectives, and, above all, through guidelines of a preventive nature that are formed to combat some aspects of psychopathology, sociopathology, and criminology [1,2].

Forensic psychiatric expertise (FPE) makes a considerable contribution to the field of clinical criminology. It is necessary to deepen interdisciplinary investigations and the tactics of evaluating and exploiting the indices of criminology and of psycho-social prognostic orientations regarding conflict, aggressiveness, adaptability, and recoverability. Interdisciplinary research should be expanded to improve evaluation methods. This includes analyzing criminological indicators and assessing psychosocial factors like conflict, aggressiveness, adaptability, and recoverability [3,4]. FPE has the function of enabling triaging and the differentiation of some potentially dangerous categories of people, with the aim of isolating them from the rest of society and establishing some protection and safety measures with the obligation of differentiated treatment, until a good remission with possibilities of social reinsertion is achieved, in order to find solutions to combat recidivism in the sense of decompensation and criminal repetition [3].

As disciplines of public health, both legal medicine and psychiatry must be leveraged jointly to realize the training of a medico-social–legal preventive nurse with the capability of acknowledging some particular aspects of psychopathology. However, prognostic assessments and the appropriate therapeutic guidelines establish appropriate safety measures, depending on the individualized interpretation of the cases [5].

Psychologically, the criteria used in forensic psychiatric examination in the United States and the United Kingdom are mainly based on cognitive traits, while in France and the Netherlands they have a broader psychological basis [6,7,8].

Forensic psychiatric expertise has a social–legal purpose, which gives it its probative value. It is derived from a synthesis of the data resulting from the examination of the scene, the injured persons, the perpetrator, and the object used. The forensic scientist, through their training and professional orientation, contributes to the legal resolution of all the mentioned elements and particularities [9]. The objectives of FPE is to enable the determination of the state of the mental health of a subject, in the context of deciding the judgement and establishing the guilt of the person who commits antisocial acts. This is based on a complex characterization of the personality of the individual being assessed, correlating ones features in relation to the psychiatric diagnosis and the constituent elements of the criminal or antisocial act committed [10].

Despite its importance, the impact of large-scale crises on FPE practices has been underexplored. Previous crises have disrupted healthcare and judicial systems, necessitating adaptations in psychiatric evaluations. Mak et al. showed that the SARS epidemic had catastrophic effects on mental health, with the most prevalent psychiatric conditions in survivors being PTSD and depressive disorders [11]. The same long-term symptoms, together with anxiety, were also reported by Cenat et al. in patients surviving the Ebola pandemic. The authors also reported a worsening of these symptoms in the context of the COVID-19 pandemic [12]. Taking into consideration the mental health challenges that have emerged in survivors of previous pandemics, such as PTSD, depression, and anxiety, it becomes evident that these psychological conditions can have far-reaching consequences if left unaddressed. Not only do they impact individual well-being, but they may also increase the risk of antisocial behaviors and, in some cases, contribute to criminal tendencies. This highlights the critical need for ongoing monitoring and targeted interventions for individuals at risk, ensuring that mental health issues are identified and managed effectively. In this context, forensic psychiatric expertise plays an essential role in addressing these risks, particularly in understanding how the COVID-19 pandemic has influenced mental health and its potential link to criminal behavior.

Prior to the COVID-19 pandemic, forensic psychiatric practices were primarily centered on in-person evaluations, where direct observation and interaction with individuals were critical to assessing mental health, judgment capacity, and criminal responsibility [13]. These evaluations typically adhered to established guidelines, such as those provided by the American Psychiatric Association (APA) and other professional bodies, ensuring standardized procedures for diagnosis and risk assessment. The process often involved comprehensive interviews, psychological testing, and collateral information from medical records, family members, and law enforcement [14,15,16]. The in-person nature of these assessments allowed for a better understanding of behavior and interpersonal dynamics, which are essential in forming accurate psychiatric diagnoses. Furthermore, forensic psychiatry before the pandemic placed a strong emphasis on courtroom testimony, where psychiatrists provided expert opinions to assist in legal decisions regarding competency, criminal intent, and recidivism risk. These practices, while effective in many respects, were challenged by logistical and ethical considerations during large-scale crises, highlighting the need for adaptive strategies in extraordinary circumstances [14,15,16,17].

The COVID-19 pandemic has underscored the importance of telepsychiatry in maintaining continuity of care while adhering to social distancing protocols and introduced significant changes to the practice of forensic psychiatry, driven by the need to adapt to restrictions on in-person interactions and the overwhelming demand on healthcare systems. Telepsychiatry emerged as a pivotal tool, allowing forensic evaluations to continue remotely through video consultations [18]. Research indicates that telepsychiatry can improve access to services, reduce costs associated with travel, and enhance the coordination of care across healthcare systems. Additionally, it has the potential to increase the number of mental health professionals willing to work in criminal justice systems and may reduce the stigma associated with receiving psychiatric services. While this shift ensured the continuity of essential services, it also posed challenges, such as the inability to observe subtle behavioral cues and the potential for compromised confidentiality in virtual settings. Courtroom procedures also evolved, with expert testimonies increasingly delivered through virtual platforms, which required adjustments in communication strategies to maintain clarity and impact [18,19,20]. Additionally, the pandemic highlighted the importance of addressing the mental health repercussions of prolonged isolation, fear, and uncertainty, both in incarcerated populations and the general public. These changes underscored the need for enhanced training in telepsychiatry, revised guidelines for remote assessments, and a renewed focus on integrating mental health care into public health crises to ensure that forensic psychiatry remains effective and resilient in the face of future challenges. Studies have demonstrated that telepsychiatric assessments are reliable, and that clinical outcomes of telepsychiatric interventions are comparable to conventional treatment among diverse patient populations, ages, and diagnostic groups. However, in many aspects of effectiveness, the evidence base is still relatively limited and often compromised by methodological problems. The lack of data on cost effectiveness, in particular, is a major hindrance, raising doubts about the continued viability of telepsychiatric services. Added to this are the vagaries of technology, negative views among clinicians, poor uptake by providers, and several legal, ethical, and administrative barriers. These hamper the widespread implementation of telepsychiatry and its integration with routine care [18,19,21].

The current study investigates the effects of the COVID-19 pandemic on forensic psychiatric expertise, analyzing variables such as demographic data (e.g., age, gender, and background) and other relevant factors, including criminal offenses, personal pathological antecedents (PPAs), diagnoses, and judgment capacity

## 2. Materials and Methods

### 2.1. Participants

An epidemiological study was conducted at the Bihor County Forensic Medicine Service between January 2021 and May 2023. A total of 186 participants were included in this study; 117 participants belonged to the “COVID group”, and 69 participants belonged to the “post-COVID group”. The COVID group refers to all patients enrolled in this study during the pandemic period, while the post-COVID group includes patients enrolled in this study after the pandemic period.

Inclusion criteria were that the participants were 18 years old at the beginning of this study and were examined by a committee consisting of a forensic pathologist and two psychiatrists with FPE. Exclusion criteria from this study were an age under 18 years old and patients who needed FPE in the civil-right context.

### 2.2. Experimental Design

The aim of this study is to compare key variables between two distinct cohorts: a COVID cohort and a post-COVID cohort. This comparison aims to elucidate the temporal changes brought about by the pandemic in terms of FPE.

This study focuses on a range of demographic variables, such as age, gender, and background, in addition to other critical factors such as criminal records, medical history, specific diagnoses, and diseases. The analytical approach involves calculating the frequencies and percentages of the variables within each cohort. The temporal dimension is also taken into consideration by comparing these variables across the pandemic and post-pandemic periods.

### 2.3. Statistical Analysis

To determine if continuous variables were normal, the Shapiro–Wilk test was employed. While non-normally distributed data were summarized using medians with interquartile ranges (IQRs; 25th to 75th percentiles), normally distributed data were expressed as means ± standard deviations (SDs). Counts and percentages were used to characterize categorical variables. When comparing two groups, Welch’s *t*-test was used to determine differences between the groups; when comparing multiple groups, one-way ANOVA was used, incorporating post hoc tests (e.g., Tukey’s HSD) to identify particular group distinctions. The Mann–Whitney U test and the Kruskal–Wallis test were used for two and multiple groups, respectively, for non-parametric continuous data. Dunn’s post hoc analyses were then performed as needed. When expected cell counts were less than five, Chi-square tests or Fisher’s exact test were used to compare the categorical data. In order to attain a statistical power of 80% and a confidence level of 95%, sample size calculations were conducted in advance using predicted effect sizes and variance estimates obtained from preliminary data. R (version 3.6) was used for all statistical analyses, making use of Finalfit for regression analysis, MCGV, Stringdist, Janitor, and Hmisc for specialized data processing requirements, and a number of extensive packages available in the Tidyverse for data manipulation and visualization.

## 3. Results

The study population comprised 186 individuals, consisting of 142 males (76%) and 44 females (24%). Participants were recruited from 2021 to 2023, with the majority enrolled in 2021 (47.8%). Of the total 186 participants, 117 (62.9%) were allocated to the COVID period group, while 69 were assigned to the post-COVID group. The mean age of the population was 43.0 years, with a range from 18 to 84 years. The women in this study were slightly older on average (46.6 years) than the men (41.9 years).

**Figure 1:**
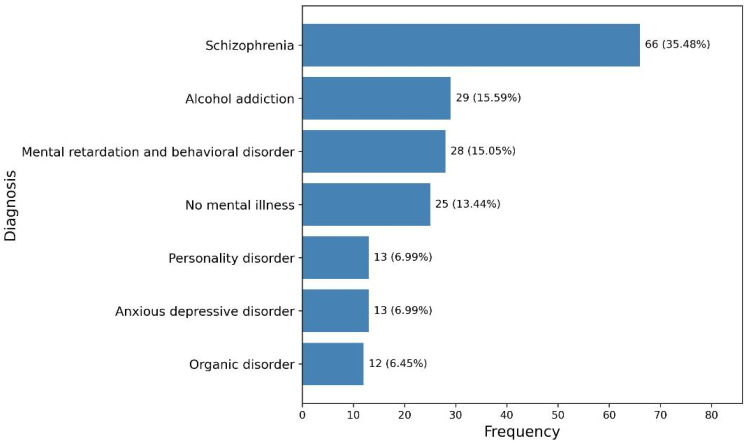
Diagnosis frequency of foresing patients.

The mean age of participants was consistent across both groups, with the COVID group having a mean age of 43.0 years (14.3) and the post-COVID group having a mean age of 43.1 years (15.6), with no significant difference (*p* = 0.9632). Regarding residence, 48.7% of the COVID group and 59.4% of the post-COVID group were from rural areas, while the remainder were from urban areas (*p* = 0.1581). When examining criminal acts, a significant difference was found (*p* = 0.0421). Aggression was the most common act in both groups (23.9% in the COVID group and 23.2% in the post-COVID group). Destruction was significantly more frequent in the post-COVID group (24.6%) compared to the COVID group (8.5%) (Figure 1). In terms of Physical and Psychiatric Assessment (PPA), while the differences were not statistically significant (*p* = 0.0821), a higher percentage of psychiatric diseases were observed in the post-COVID group (60.9%) compared to the COVID group (51.3%). For forensic history, a higher percentage of the COVID group had a forensic history (33.3%) compared to the post-COVID group (21.7%) (*p* = 0.0921).

Schizophrenia was the most common diagnosis in both groups (24.8% in the COVID group and 26.1% in the post-COVID group). Hospitalization at the time of examination showed a significant difference (*p* = 0.0041), with a higher percentage of the COVID group hospitalized (37.6%) compared to the post-COVID group (17.4%).

In terms of diseases, the prevalence was similar in both groups, with 83.8% in the COVID group and 79.7% in the post-COVID group, and this difference was not statistically significant (*p* = 0.4851). Finally, the level of discernment showed no significant difference between the groups (*p* = 0.6131). Present discernment was observed in 45.3% of the COVID group and 52.2% of the post-COVID group.

Safety measures were applied similarly across both groups, with no significant differences (*p* = 0.1291). The majority of participants in both groups did not require any safety measures (Table 1).

A statistically significant difference was found between the rural and urban distribution (*p* = 0.0051). Most male participants (58.5%) were from rural areas, while a higher proportion of females (65.9%) were from urban environments.

Regarding criminal offenses, aggression was the most common crime, affecting 23.7% of the total population. Notably, sexual assault was more frequent among women (15.9% compared to 4.9% of men). Additionally, other offenses, including child pornography, were more prevalent among women (27.3% vs. 18.3% for men). The data showed a significant gender difference in legal outcomes, particularly with mandatory hospitalization, which was significantly higher in women (43.2%) compared to men (16.2%, *p* < 0.0011).

In terms of health, the presence of somatic or mental illnesses (APP) was similar across genders, with over half of the participants suffering from mental illnesses (54.8%). However, women were more likely to suffer from both somatic and mental conditions (27.3%) compared to men (19.7%).

There was a significant difference in discernment between men and women (*p* = 0.0051). More women (56.8%) were found to lack discernment, while only 30.3% of men had absent discernment. This difference extended into legal outcomes, where women had a higher proportion of mandatory hospitalization (43.2%) compared to men (16.2%) (Table 2).

We conducted an analysis of the 186 patients who were divided into three groups based on their judgement: 89 with preserved judgement, 68 with no judgement, and 29 with impaired judgement. The majority of patients (62.9%) were from the COVID cohort, while the remaining 37.1% were from the post-COVID cohort. The mean age of all groups was 43.0 years (range: 18–84 years), with no significant age differences between the differentiating groups.

There was a significant difference in gender distribution (*p* = 0.0051). Males made up the majority of the preserved and diminished discernment groups (85.4% and 79.3%, respectively), whereas the absent discernment group had a higher proportion of females (36.8%).

No significant differences were observed regarding the rural–urban distribution across the discernment groups (*p* = 0.9471). Approximately 52.7% of the total population lived in rural areas, with similar proportions in all discernment categories.

The types of criminal offenses committed varied across the discernment groups. Notably, aggression was more frequent among patients with absent discernment (33.8%), compared to 14.6% in the preserved group and 27.6% in the diminished group. Additionally, 11.2% of those with preserved discernment were involved in attempted murder, compared to 20.7% in the diminished group.

There was a significant association between discernment and the presence of both somatic and mental illnesses (*p* < 0.0011). Patients with absent discernment had the highest prevalence of mental illnesses (67.6%), followed by 47.2% in the preserved group. Interestingly, 31.5% of those with preserved discernment had no history of medical or psychological illness, compared to only 1.5% in the absent group.

A significant difference was observed in the application of safety measures (*p* < 0.0011). The majority of patients with absent discernment (60.3%) were subject to mandatory hospitalization (Art. 110), whereas 94.4% of those with preserved discernment required no such measures.

There was a significant variation in age distribution (*p* = 0.0121). The largest age group in the study was the 25–44 years (44.1%), but younger patients (18–24 years) were more prevalent in the preserved and diminished discernment groups (16.9% and 17.2%, respectively) (Table 3).

## 4. Discussion

In forensic psychiatric evaluations, particularly when the focus extends to criminological aspects, discussions have sometimes moved beyond the concrete determinism of biopsychological phenomena to encompass social life. This shift can create challenges in translating causal relationships into legal terms [11]. More often, these discussions have been overlooked, with the primary focus being on providing forensic criteria for legal responsibility, particularly regarding the degree of discernment as an expression of consciousness [12,13]. The causal link between a mental diagnosis and an antisocial act is often implied or indirectly expressed [14]. However, this approach is often overly simplistic, especially when it is limited to the context of the examination or when it focuses solely on the relationship between psychiatric diagnosis and discernment or between the minor’s age and discernment [14,15,16,17].

The challenges arising from such an approach to translating the forensic “equivalents” of responsibility for judicial purposes are attributed to the current criteria. These criteria are often seen as formal and arbitrary and fail to adequately quantify the individualization required for sentencing [18,19,20,21,22].

Forensic investigation sometimes extends to the individual who acted, considering their complex biological, psychological, and social functions, especially when the judiciary is concerned with their mental state [23,24]. In a criminal case, the pursuit of truth belongs to the judiciary, with forensic medicine contributing particularly to the physical, biological, and psychological domains [11,13,14,18].

In interpreting the determinism of complex phenomena where biological, psychological, and social factors intertwine, with each being uniquely influenced by individual personality, it is essential to investigate the cause of somato-psychic changes within the context of interaction [25]. This interaction often manifests as the interdependence between a system’s internal and external factors [25,26], highlighting the triggering role of both external and internal factors in different situations. The challenge in forensic evaluation and research lies in objectively identifying these factors [27].

Determining an individual’s capacity for critical assessment of the content and, particularly, the consequences of an antisocial act, provides evidence of their level of consciousness concerning the imputable act [16,17,26,28]. As previously discussed, demonstrating the causal link between the perpetrator’s personality traits and the elements constituting the antisocial act committed is crucial in establishing responsibility.

In our study, statistical data were collected over three years (2021–2023) and categorized based on the pandemic and post-pandemic periods. We consistently recorded a higher percentage of male participants across all three years, without noting significant differences based on their background.

One of the key findings was the higher prevalence of aggression and child pornography cases during the COVID-19 pandemic compared to the post-pandemic period. These results align with the existing literature, suggesting that periods of social isolation, increased stress, and restricted access to support systems during the pandemic contributed to a rise in violent and abusive behaviors. Pandemic-induced disruptions, such as prolonged lockdowns, financial insecurity, and social isolation, likely exacerbated underlying psychological vulnerabilities, increasing the propensity for antisocial behaviors in certain individuals [11].

Another significant observation was the elevated prevalence of psychiatric disorders during the COVID-19 period, often in connection to somatic illnesses. Previous studies have documented the psychological toll of the pandemic, including higher rates of anxiety, depression, and substance use disorders among the general population [25]. Our data further corroborate these findings, highlighting the comorbid nature of psychiatric and somatic conditions during the pandemic. The stress of the pandemic may have acted as a trigger for the worsening of pre-existing mental health conditions or the emergence of new disorders, potentially explaining the higher rates observed in our study. D’Orta et al. emphasized in their study that the COVID-19 pandemic was linked to an increased demand for acute forensic care, particularly among detainees with personality disorders that involved increased impulsivity, lower frustration tolerance, loss of control, high extraversion, and a high prevalence of substance use disorder comorbidity. In forensic contexts, telepsychiatry has been utilized for various applications, including evaluations and consultations. While the research supporting the use of telepsychiatry for forensic evaluations is not as extensive as that for clinical applications, there are encouraging findings. For instance, studies have found high levels of agreement between in-person and remote telepsychiatry-based assessments for competency to stand trial. These findings suggest that telepsychiatric assessments can achieve results equivalent to traditional, in-person evaluations [27].

In terms of psychiatric disorder and substance use disorders, an increased frequency of schizophrenia and alcohol dependency during the pandemic was observed [29]. Schizophrenia is known to be aggravated by stress and social isolation [30]. Also, some studies show that COVID-19-related mortality during pandemics seems to be higher in people with schizophrenia. Also, alcohol dependency could be attributed to the use of alcohol as a coping mechanism during periods of high anxiety and limited access to mental health support [29]. These findings suggest that public health measures aimed at reducing the psychological impact of future crises should include targeted interventions for vulnerable populations.

Moreover, our data indicate that discernment and the need for security measures were more prevalent during the pandemic. This trend could reflect high social tension and a corresponding increase in the perceived risk of harm. This raises the necessity of stricter forensic evaluations and security protocols. The pandemic’s unique stressors may have impaired individuals’ capacity for critical judgment, thereby influencing their criminal behavior and requiring enhanced interventions during forensic assessments [31].

Interestingly, while the criminal acts recorded during the pandemic showed higher percentages of aggression and child pornography, the post-pandemic period exhibited a decline in these categories. This decrease may be attributed to the restoration of social structures, reopening of institutions, and improved access to mental health care, which collectively contributed to a stabilization of behaviors. However, the persistence of certain psychiatric disorders, even post-pandemic, emphasize the need for sustained mental health support.

This study provides novel medico-legal and socio-psychological criteria for understanding how large-scale crises, such as the COVID-19 pandemic, influence criminal behavior and mental health in forensic contexts. By analyzing demographic, diagnostic, and behavioral data, this research highlights the connection between external stressors and individual vulnerabilities in shaping antisocial behaviors. The findings also emphasize the critical role of forensic psychiatry in adapting to emerging challenges during and after public health emergencies. One significant adaptation during the pandemic has been the integration of telepsychiatry, which allowed continued patient care while maintaining social distancing protocols. As the pandemic subsides, it is important to consider whether telepsychiatry will remain a viable option in forensic settings. Potential benefits include increased accessibility for patients in remote areas, reduced costs, and the ability to conduct evaluations more efficiently.

## 5. Limitations of the Study

The limitations of this study include its retrospective design, which may introduce recall bias and limit control over confounding variables; the relatively small sample size, which may not detect smaller but significant differences between subgroups; and the fact that data were collected from a single forensic medicine service, limiting the generalizability of findings to other regions or institutions. Additionally, the lack of long-term follow-up hinders the understanding of treatment efficacy or judicial outcomes, while potential under-reporting of offenses like child pornography and sexual harassment could skew crime prevalence rates. The influence of pandemic-related factors may have impacted psychiatric assessments and behavior, complicating the isolation of forensic psychiatric evaluation effects, and the absence of detailed socioeconomic data further limits insight into the broader influences on criminal behavior and psychiatric disorders. These limitations underscore the need for caution when generalizing this study’s findings and suggest areas for future research. Further research is necessary to explore the long-term effects of the pandemic on forensic psychiatric evaluations and to validate these findings in larger, more diverse populations. Future studies should also investigate the mechanisms underlying the observed trends, particularly the relationship between stress-induced psychiatric disorders and criminal behavior. Developing robust, adaptable forensic protocols for future crises will be essential to ensure accurate evaluations and effective interventions.

## 6. Conclusions

The investigation and treatment of psychiatric patients are critical components of criminal law, particularly within European Union countries, where psychiatric evaluations play a vital role in criminal justice and the rehabilitation of offenders. Our analysis reveals no significant increase in the frequency of the studied objectives during the pandemic compared to the post-pandemic period. However, higher percentages of aggression, child pornography cases, and attempted murder were observed during the COVID-19 period. Additionally, psychiatric disorders were more prevalent, often co-occurring with somatic illnesses and legal issues. Importantly, security measures were more frequently required during the pandemic than in the post-pandemic period.

To address these challenges, it is recommended that policy adjustments be made to enhance the capacity for remote psychiatric evaluations, ensuring that they do not compromise the quality of care. Staff training on conducting remote assessments should be prioritized, with a particular focus on managing complex cases that involve comorbidities. Furthermore, future research should explore the long-term impact of pandemic-related changes on the accuracy and effectiveness of psychiatric evaluations and examine how remote assessments perform across different patient demographics.

## Figures and Tables

**Table 1:** Distribution of demographic variables.

	COVID Group (N = 117)	Post COVID Group (N = 69)	Total (N = 186)	*p* Value
**Gender**				0.809 ^1^
Male	90.0 (76.9%)	52.0 (75.4%)	142.0 (76.3%)	
Female	27.0 (23.1%)	17.0 (24.6%)	44.0 (23.7%)	
**Year**				<0.001 ^1^
2021	89.0 (76.1%)	0.0 (0.0%)	89.0 (47.8%)	
2022	28.0 (23.9%)	43.0 (62.3%)	71.0 (38.2%)	
2023	0.0 (0.0%)	26.0 (37.7%)	26.0 (14.0%)	
**Age**				0.963 ^2^
Mean (SD)	43.0 (14.3)	43.1 (15.6)	43.0 (14.7)	
Range	21.0–84.0	18.0–76.0	18.0–84.0	
**Residence**				0.158 ^1^
Rural	57.0 (48.7%)	41.0 (59.4%)	98.0 (52.7%)	
Urban	60.0 (51.3%)	28.0 (40.6%)	88.0 (47.3%)	
**Criminal act**				0.042 ^1^
Aggression	28.0 (23.9%)	16.0 (23.2%)	44.0 (23.7%)	
Menace	9.0 (7.7%)	0.0 (0.0%)	9.0 (4.8%)	
Homicide	4.0 (3.4%)	2.0 (2.9%)	6.0 (3.2%)	
Sexual Harassment	7.0 (6.0%)	7.0 (10.1%)	14.0 (7.5%)	
Fraud	2.0 (1.7%)	0.0 (0.0%)	2.0 (1.1%)	
Other (Infant Pornography, etc.)	26.0 (22.2%)	12.0 (17.4%)	38.0 (20.4%)	
Destruction	10.0 (8.5%)	17.0 (24.6%)	27.0 (14.5%)	
Attempted Murder	16.0 (13.7%)	7.0 (10.1%)	23.0 (12.4%)	
Statutory Rape	4.0 (3.4%)	4.0 (5.8%)	8.0 (4.3%)	
Abandonment	11.0 (9.4%)	4.0 (5.8%)	15.0 (8.1%)	
**PPA**				0.082 ^1^
Somatic Diseases	10.0 (8.5%)	2.0 (2.9%)	12.0 (6.5%)	
Psychiatric Diseases	60.0 (51.3%)	42.0 (60.9%)	102.0 (54.8%)	
Both	30.0 (25.6%)	10.0 (14.5%)	40.0 (21.5%)	
None	17.0 (14.5%)	15.0 (21.7%)	32.0 (17.2%)	
**Forensic history**				0.092 ^1^
Yes	39.0 (33.3%)	15.0 (21.7%)	54.0 (29.0%)	
No	78.0 (66.7%)	54.0 (78.3%)	132.0 (71.0%)	
**Hospitalization at the time of examination**				0.004 ^1^
Yes	44.0 (37.6%)	12.0 (17.4%)	56.0 (30.1%)	
No	73.0 (62.4%)	57.0 (82.6%)	130.0 (69.9%)	
**Diseases**				0.485^1^
Present	98.0 (83.8%)	55.0 (79.7%)	153.0 (82.3%)	
Absent	19.0 (16.2%)	14.0 (20.3%)	33.0 (17.7%)	
**Discernment**				0.613 ^1^
Present	53.0 (45.3%)	36.0 (52.2%)	89.0 (47.8%)	
Absent	44.0 (37.6%)	24.0 (34.8%)	68.0 (36.6%)	
Diminished	20.0 (17.1%)	9.0 (13.0%)	29.0 (15.6%)	
**Safety measures**				0.129 ^1^
No expert needed	6.0 (5.1%)	6.0 (8.7%)	12.0 (6.5%)	
Free with compulsory treatment (art 109)	24.0 (20.5%)	8.0 (11.6%)	32.0 (17.2%)	
Mandatory hospitalization (art 110)	30.0 (25.6%)	12.0 (17.4%)	42.0 (22.6%)	
None needed	57.0 (48.7%)	43.0 (62.3%)	100.0 (53.8%)	

^1^ Pearson chi square, ^2^ Linear model anova.

**Table 2:** Gender differences in criminal behavior, mental health, and legal outcome.

	Male (N = 142)	Female (N = 44)	Total (N = 186)	*p* Value
**Year**				0.510 ^1^
2021	70.0 (49.3%)	19.0 (43.2%)	89.0 (47.8%)	
2022	51.0 (35.9%)	20.0 (45.5%)	71.0 (38.2%)	
2023	21.0 (14.8%)	5.0 (11.4%)	26.0 (14.0%)	
**Age**				0.062 ^2^
Mean (SD)	41.9 (14.6)	46.6 (14.8)	43.0 (14.7)	
Range	18.0–76.0	20.0–84.0	18.0–84.0	
**Residence**				0.005 ^1^
Rural area	83.0 (58.5%)	15.0 (34.1%)	98.0 (52.7%)	
Urban area	59.0 (41.5%)	29.0 (65.9%)	88.0 (47.3%)	
**Criminal act**				0.019 ^1^
Aggression	33.0 (23.2%)	11.0 (25.0%)	44.0 (23.7%)	
Menace	9.0 (6.3%)	0.0 (0.0%)	9.0 (4.8%)	
Homicide	4.0 (2.8%)	2.0 (4.5%)	6.0 (3.2%)	
Sexual Harassment	7.0 (4.9%)	7.0 (15.9%)	14.0 (7.5%)	
Fraud	2.0 (1.4%)	0.0 (0.0%)	2.0 (1.1%)	
Other (Infant Pornography, etc.)	26.0 (18.3%)	12.0 (27.3%)	38.0 (20.4%)	
Destruction	23.0 (16.2%)	4.0 (9.1%)	27.0 (14.5%)	
Attempted Murder	19.0 (13.4%)	4.0 (9.1%)	23.0 (12.4%)	
I confirm	4.0 (2.8%)	4.0 (9.1%)	8.0 (4.3%)	
Abandonment	15.0 (10.6%)	0.0 (0.0%)	15.0 (8.1%)	
**PPA**				0.650 ^1^
Somatic Diseases	10.0 (7.0%)	2.0 (4.5%)	12.0 (6.5%)	
Psychiatric Diseases	78.0 (54.9%)	24.0 (54.5%)	102.0 (54.8%)	
Both	28.0 (19.7%)	12.0 (27.3%)	40.0 (21.5%)	
None	26.0 (18.3%)	6.0 (13.6%)	32.0 (17.2%)	
**Forensic history**				0.641 ^1^
Yes	40.0 (28.2%)	14.0 (31.8%)	54.0 (29.0%)	
No	102.0 (71.8%)	30.0 (68.2%)	132.0 (71.0%)	
**Hospitalization at the time of examination**				0.301 ^1^
Yes	40.0 (28.2%)	16.0 (36.4%)	56.0 (30.1%)	
No	102.0 (71.8%)	28.0 (63.6%)	130.0 (69.9%)	
**Disease**				0.415 ^1^
Present	115.0 (81.0%)	38.0 (86.4%)	153.0 (82.3%)	
Absent	27.0 (19.0%)	6.0 (13.6%)	33.0 (17.7%)	
**Discernment**				0.005 ^1^
Present	76.0 (53.5%)	13.0 (29.5%)	89.0 (47.8%)	
Absent	43.0 (30.3%)	25.0 (56.8%)	68.0 (36.6%)	
Diminished	23.0 (16.2%)	6.0 (13.6%)	29.0 (15.6%)	
**Safety measures**				<0.001 ^1^
No expert needed	7.0 (4.9%)	5.0 (11.4%)	12.0 (6.5%)	
Free with compulsory treatment (art 109)	29.0 (20.4%)	3.0 (6.8%)	32.0 (17.2%)	
Mandatory hospitalization (art 110)	23.0 (16.2%)	19.0 (43.2%)	42.0 (22.6%)	
None needed	83.0 (58.5%)	17.0 (38.6%)	100.0 (53.8%)	

^1^ Pearson’s Chi-squared test, ^2^ Linear Model ANOVA.

**Table 3:** Demographic, clinical, and forensic characteristics of participants stratified by discernment.

	Present (N = 89)	Absent (N = 68)	Diminished (N = 29)	Total (N = 186)	*p* Value
**Age**					0.012 ^1^
18–24 years (early adulthood)	15.0 (16.9%)	2.0 (2.9%)	5.0 (17.2%)	22.0 (11.8%)	
25–44 years (prime working age)	35.0 (39.3%)	37.0 (54.4%)	10.0 (34.5%)	82.0 (44.1%)	
45–64 years (pre-retirement)	34.0 (38.2%)	20.0 (29.4%)	8.0 (27.6%)	62.0 (33.3%)	
65+ years (retirement and beyond)	5.0 (5.6%)	9.0 (13.2%)	6.0 (20.7%)	20.0 (10.8%)	
**Group**					0.613 ^1^
COVID	53.0 (59.6%)	44.0 (64.7%)	20.0 (69.0%)	117.0 (62.9%)	
Post-COVID	36.0 (40.4%)	24.0 (35.3%)	9.0 (31.0%)	69.0 (37.1%)	
**Year**					0.175 ^1^
2021	35.0 (39.3%)	37.0 (54.4%)	17.0 (58.6%)	89.0 (47.8%)	
2022	38.0 (42.7%)	25.0 (36.8%)	8.0 (27.6%)	71.0 (38.2%)	
2023	16.0 (18.0%)	6.0 (8.8%)	4.0 (13.8%)	26.0 (14.0%)	
**Gender**					0.005 ^1^
Male	76.0 (85.4%)	43.0 (63.2%)	23.0 (79.3%)	142.0 (76.3%)	
Female	13.0 (14.6%)	25.0 (36.8%)	6.0 (20.7%)	44.0 (23.7%)	
**Residence**					0.947 ^1^
Rural	46.0 (51.7%)	36.0 (52.9%)	16.0 (55.2%)	98.0 (52.7%)	
Urban	43.0 (48.3%)	32.0 (47.1%)	13.0 (44.8%)	88.0 (47.3%)	
**Criminal act**					0.071 ^1^
Aggression	13.0 (14.6%)	23.0 (33.8%)	8.0 (27.6%)	44.0 (23.7%)	
Menace	6.0 (6.7%)	3.0 (4.4%)	0.0 (0.0%)	9.0 (4.8%)	
Homicide	6.0 (6.7%)	0.0 (0.0%)	0.0 (0.0%)	6.0 (3.2%)	
Sexual Harassment	6.0 (6.7%)	6.0 (8.8%)	2.0 (6.9%)	14.0 (7.5%)	
Fraud	0.0 (0.0%)	1.0 (1.5%)	1.0 (3.4%)	2.0 (1.1%)	
Other (Infant Pornography, etc.)	18.0 (20.2%)	13.0 (19.1%)	7.0 (24.1%)	38.0 (20.4%)	
Destruction	13.0 (14.6%)	11.0 (16.2%)	3.0 (10.3%)	27.0 (14.5%)	
Attempted Murder	10.0 (11.2%)	7.0 (10.3%)	6.0 (20.7%)	23.0 (12.4%)	
Statutory Rape	6.0 (6.7%)	2.0 (2.9%)	0.0 (0.0%)	8.0 (4.3%)	
Abandonment	11.0 (12.4%)	2.0 (2.9%)	2.0 (6.9%)	15.0 (8.1%)	
**PPA**					<0.001 ^1^
Somatic Diseases	6.0 (6.7%)	4.0 (5.9%)	2.0 (6.9%)	12.0 (6.5%)	
Psychiatric Diseases	42.0 (47.2%)	46.0 (67.6%)	14.0 (48.3%)	102.0 (54.8%)	
Both	13.0 (14.6%)	17.0 (25.0%)	10.0 (34.5%)	40.0 (21.5%)	
None	28.0 (31.5%)	1.0 (1.5%)	3.0 (10.3%)	32.0 (17.2%)	
**Forensic history**					0.510^1^
Yes	26.0 (29.2%)	22.0 (32.4%)	6.0 (20.7%)	54.0 (29.0%)	
No	63.0 (70.8%)	46.0 (67.6%)	23.0 (79.3%)	132.0 (71.0%)	
**Diagnostic**					<0.001 ^1^
Schizophrenia	21.0 (23.6%)	38.0 (55.9%)	7.0 (24.1%)	66.0 (35.5%)	
Dissociative Paranoid Disorder	2.0 (2.2%)	5.0 (7.4%)	5.0 (17.2%)	12.0 (6.5%)	
Organic Disorder	25.0 (28.1%)	0.0 (0.0%)	0.0 (0.0%)	25.0 (13.4%)	
No psychiatric Diseases	22.0 (24.7%)	4.0 (5.9%)	3.0 (10.3%)	29.0 (15.6%)	
Alcohol Addiction	4.0 (4.5%)	8.0 (11.8%)	1.0 (3.4%)	13.0 (7.0%)	
Anxiety–Depression Disorder	3.0 (3.4%)	6.0 (8.8%)	4.0 (13.8%)	13.0 (7.0%)	
Personality Disorder	12.0 (13.5%)	7.0 (10.3%)	9.0 (31.0%)	28.0 (15.1%)	
**Hospitalization at the time of examination**					<0.001 ^1^
Yes	21.0 (23.6%)	32.0 (47.1%)	3.0 (10.3%)	56.0 (30.1%)	
No	68.0 (76.4%)	36.0 (52.9%)	26.0 (89.7%)	130.0 (69.9%)	
**Diseases**					<0.001 ^1^
Present	56.0 (62.9%)	68.0 (100.0%)	29.0 (100.0%)	153.0 (82.3%)	
Absent	33.0 (37.1%)	0.0 (0.0%)	0.0 (0.0%)	33.0 (17.7%)	
**Safety measures**					<0.001 ^1^
No expert needed	1.0 (1.1%)	7.0 (10.3%)	4.0 (13.8%)	12.0 (6.5%)	
Free with compulsory treatment (art 109)	4.0 (4.5%)	16.0 (23.5%)	12.0 (41.4%)	32.0 (17.2%)	
Mandatory hospitalization (art 110)	0.0 (0.0%)	41.0 (60.3%)	1.0 (3.4%)	42.0 (22.6%)	
None needed	84.0 (94.4%)	4.0 (5.9%)	12.0 (41.4%)	100.0 (53.8%)	

^1^ Pearson chi square.

## Data Availability

The original contributions presented in this study are included in the article. Further inquiries can be directed to the corresponding author.
